# Persistence of *Plasmodium falciparum* HRP-2 antigenaemia after artemisinin combination therapy is not associated with gametocytes

**DOI:** 10.1186/s12936-022-04387-0

**Published:** 2022-12-06

**Authors:** Tate Oulton, Almahamoudou Mahamar, Koualy Sanogo, Makonon Diallo, Ahamadou Youssouf, Sidi M. Niambele, Siaka Samaké, Sekouba Keita, Youssouf Sinaba, Adama Sacko, Sekou F. Traore, Kjerstin Lanke, Katharine A. Collins, John Bradley, Chris Drakeley, Will J. R. Stone, Alassane Dicko

**Affiliations:** 1 Department of Infection Biology, London School of Hygiene & Tropical Medicine, London, UK; 2 Malaria Research and Training Centre, Faculty of Pharmacy and Faculty of Medicine and Dentistry, University of Sciences Techniques and Technologies of Bamako, Bamako, Mali; 3 Department of Medical Microbiology and Radboud Center for Infectious Diseases, Radboud University Medical Center, University of Nijmegen, Nijmegen, The Netherlands; 4 MRC International Statistics and Epidemiology Group, London School of Hygiene and Tropical Medicine, London, UK

**Keywords:** Malaria, Rapid diagnostic tests, Lateral flow, Ultra-sensitive RDT, HRP-2, Gametocytes, Antigenaemia, Infectiousness

## Abstract

**Background:**

In some settings, sensitive field diagnostic tools may be needed to achieve elimination of falciparum malaria. To this end, rapid diagnostic tests (RDTs) based on the detection of the *Plasmodium falciparum* protein HRP-2 are being developed with increasingly lower limits of detection. However, it is currently unclear how parasite stages that are unaffected by standard drug treatments may contribute to HRP-2 detectability and potentially confound RDT results even after clearance of blood stage infection. This study assessed the detectability of HRP-2 in periods of post-treatment residual gametocytaemia.

**Methods:**

A cohort of 100 *P.* *falciparum* infected, gametocyte positive individuals were treated with or without the gametocytocidal drug primaquine (PQ), alongside standard artemisinin-based combination therapy (ACT), in the context of a randomised clinical trial in Ouelessebougou, Mali. A quantitative ELISA was used to measure levels of HRP-2, and compared time to test negativity using a standard and ultra-sensitive RDT (uRDT) between residual gametocyte positive and negative groups.

**Results:**

Time to test negativity was longest by uRDT, followed by ELISA and then standard RDT. No significant difference in time to negativity was found between the treatment groups with and without residual gametocytes: uRDT (HR 0.79 [95% CI 0.52–1.21], p = 0.28), RDT (HR 0.77 [95% CI 0.51–1.15], p = 0.20) or ELISA (HR 0.88 [95% CI 0.59–1.32], p = 0.53). Similarly, no difference was observed when adjusting for baseline asexual parasite density. Quantified levels of HRP-2 over time were similar between groups, with differences attributable to asexual parasite densities. Furthermore, no difference in levels of HRP-2 was found between individuals who were or were not infectious to mosquitoes (OR 1.19 [95% CI 0.98–1.46], p = 0.077).

**Conclusions:**

Surviving sexual stage parasites after standard ACT treatment do not contribute to the persistence of HRP-2 antigenaemia, and appear to have little impact on RDT results.

**Supplementary Information:**

The online version contains supplementary material available at 10.1186/s12936-022-04387-0.

## Background

Historically, diagnosis of malaria infection has been dependent on a visual detection of blood stage parasites by blood film microscopy. However, the 2011 Malaria Eradication Research Agenda (malERA) process concluded that novel and improved tools are needed to detect and target transmission at its lowest levels for use in elimination contexts. This conclusion stemmed from the growing body of evidence that submicroscopic parasite densities were capable of maintaining a viable reservoir of infection in humans [1]. Although previous elimination successes have been achieved with little focus on low density or asymptomatic cases, persistence of malaria in areas even with high coverage of control measures and treatment strongly suggests that improved surveillance and targeting of the transmission reservoir is necessary to achieve elimination more widely [2].

Earlier innovations in the 00's led to the development of immuno-assay based tools, which facilitate parasite detection in blood quickly and cheaply. Such rapid diagnostic tests (RDTs) are primarily based on the capture of *Plasmodium* protein antigens in a lateral flow assay format. Current RDTs are based on the detection of a select combination of secreted parasite proteins that allow some species differentiation such as lactate dehydrogenase and aldolase; for *Plasmodium falciparum* the most commonly used is histidine rich protein-2 (HRP-2) [3–5].

HRP-2 is produced and secreted by all asexual parasites and developing sexual-stage gametocytes, and is known to be localised within mature gametocytes [6, 7]. Thus, HRP-2 is highly abundant in the blood during infection. Probability of detection will be a function of parasite density and the duration of infection, but even relatively low-density infections (200 parasites/μl) produce levels of HRP-2 that are reliably detectable by RDT [8]; the lower limit of detection typically falling between 600 and 1000 pg protein/mL [9]. In line with the recommendations made by malERA, developments in the design of HRP-2 based malaria RDTs has led to greatly increased sensitivity; these ultra-sensitive RDTs (uRDT) may detect as little as ~80 pg/mL [9, 10]. Such improvements in sensitivity are valuable in the context of enhanced detection of infection in endemic settings, particularly when lower density, asymptomatic infections are prevalent [1].

However, detectable HRP-2 will persist in circulation even after live parasite clearance following treatment. Attempts to derive parasite densities based on quantification of HRP-2 [10] are confounded by this stability of HRP-2 protein in the blood; in a study of individuals from Angola, Senegal and Tanzania, the half-life of HRP-2 in the blood was demonstrated to be 3.0–4.7 days [11]. It is understood that time to HRP-2 RDT negativity is thus largely determined by factors including peak parasite density and genetic variation in HRP-2 expression. Differences in HRP-2 expression by parasite life cycle stage may also be involved, though it is currently unclear as to if, and how significantly, mature sexual stage parasites contribute to circulating levels of HRP-2.

The importance of this question is specific to *P. falciparum*, the only human parasite to express HRP-2. *Plasmodium falciparum* gametocytes can persist for weeks after resolution of asexual infection following treatment with standard artemisinin-based combination therapy (ACT), which is incompletely effective against mature gametocytes [12, 13]. Though the exact mechanisms and efficacy of the gametocytocidal activity of ACT are unclear at present, the sterilising and gametocytocidal activities of the drug primaquine are well recognised [14, 15]; the World Health Organization (WHO) policy recommends a single dose of primaquine (PQ) with ACT for *P. falciparum* malaria in low transmission areas to further reduce risk of transmission [16]. As such, any significant contribution of HRP-2 by surviving sexual stages after ACT without PQ may affect estimates of RDT positivity particularly from uRDTs. Similarly, an understanding of gametocyte related HRP-2 levels may provide insights into post treatment infectivity, where gametocytes that survive continue to render an individual infectious to mosquitoes, even in the absence of asexual parasites [12, 17, 18].

In this study, HRP-2 dynamics were quantified in a cohort of *P. falciparum* infected, gametocyte positive Malian individuals treated with an ACT (either pyronaridine-artesunate or dihydroartemisinin-piperaquine) with and without a single low dose of the gametocidal drug primaquine to rapidly clear gametocytes. This study allows us to evaluate the specific contribution of the post-treatment gametocytes to HRP-2 levels after asexual clearance. Additionally, quantitation of HRP-2 was compared to standard and ultra-sensitive RDT and, through the determination of individual infectivity to mosquitoes by membrane feeding assays, the association between transmission potential and measurable HRP-2 was examined.

## Methods

### Study location and sample collection

The study was carried out in Ouelessebougou, Mali, in a trial conducted between September and January 2020. 100 asymptomatic individuals aged 5–50 years (inclusive), with a sexual stage density of ≥ 16 gametocytes/μL, were recruited and randomly assigned to one of four treatment arms: pyronaridine-artesunate (PA) (n = 25), PA plus single low dose primaquine (PA-PQ) (n = 25), dihydroartemisinin-piperaquine (DP) (n = 25), and DP plus single low dose primaquine (DP-PQ) (n = 25). All participants were followed for 49 days post initial treatment, receiving a full clinical and parasitological examination (including asexual and sexual stage parasite density measures by microscopy [asexual at baseline only] and qRT-PCR) on days 1, 2, 7, 14, 21, 28, 35, 42 and 49 (to day 28 only in PQ treated groups). A subsequent dose of DP at day 21 was given to ensure continued prophylaxis for the duration of the study.

### Parasite quantification

Individuals were recruited to the trial on the basis of blood smear examination and gametocyte quantification, but ring stages and gametocytes were subsequently quantified by highly sensitive qRT-PCR, as described [14]. Briefly, EDTA blood (EDTA VACUETTE tube, Greiner Bio-One, Kremsmünster, Austria) was aliquoted into RNA protect cell reagent (Qiagen, Hilden, Germany) and stored at − 80 °C until temperature tracked shipment on dry ice to Radboud University Medical Center (Nijmegen, Netherlands) for assay performance. Total nucleic acids were extracted using a MagNAPure LC automated extractor (Total Nucleic Acid Isolation Kit-High Performance; Roche Applied Science, Indianapolis, IN, USA). Male and female gametocytes were quantified in a multiplex reverse transcriptase quantitative PCR (RT-qPCR) assay as described [14]. Asexual stages were quantified by quantification of SBP1 transcripts, as described elsewhere [19]. Samples were classified as negative for a particular gametocyte sex if the qRT-PCR quantified density of gametocytes of that sex was less than 0·01 gametocytes per μL (i.e. one gametocyte per 100 μL of blood sample).

### Rapid diagnostic testing

Two rapid diagnostic tests were utilised in this study analysis: the Standard Diagnostics, Inc. (SD) BIOLINE Malaria Ag P.f. RDT (SD/Alere, Yongin-si, Republic of Korea, Cat. 05EK50), the Alere Malaria Ag P.f RDT ULTRA SENSITIVE (SD/Alere, Yongin-si, Republic of Korea). Each are based on the detection of HRP-2 and was used according to the manufacturers instruction. All Standard RDTs were performed at the same day where the blood was collected. The Alere Malaria Ag P.f RDT ULTRA SENSITIVE was performed on the same day as blood collection for all participants until day 21. All other tests were completed retrospectively using the frozen EDTA sample. For both RDTs, results were read and recorded separately by two laboratory technicians and in case of discrepancy the result was given by a third technician.

### HRP-2 quantification

Levels of HRP-2 were determined using the Quansys Q-plex Human Malaria (5-plex) kit, Q-View Imager LS and Q-View software v3.13 (Quansys Biosciences, Logan, UT, USA). The assay was carried out according to the protocol and with reagents supplied. Briefly, neat serum samples were pre-diluted 2:5 in calibrator diluent, and subsequently diluted 1:4 in complete assay diluent for a final dilution of 1:10. The assay calibration curve was prepared as directed. 50 μL of prepared sample and duplicate wells of calibration curve material were added to the plate and incubated on a shaker at 500 RPM for 2 h at room temperature (RT). Plates were washed three times using an automated microplate washer (BioTek ELx405, Agilent, Santa Clara, CA, USA) before 50 μL of detection mix was added to each well and subsequently incubated for 1 h at 500 RPM RT. Plates were then washed 3 times and 50 μL of provided Streptavidin-HRP was added, and incubated for 30 min at 500RPM RT. Plates were then washed 6 times before 50 μL of prepared substrate was added. Plates were imaged within 5 min of the addition of substrate, using an exposure time of 270 s and standard image processing. Image processing and analysis was carried out within the Q-View software, with the quantified calibration curve generated using the inbuilt 5-parameter logistic regression model.

### Mosquito feeding

Mosquito feeding was carried out as previously described [14]. Approximately 70 locally reared Anopheles gambiae were allowed to feed for 15–20 min on venous blood samples (Lithium Heparin VACUETTE tube; Greiner Bio-One, Kremsmünster, Austria) through a prewarmed glass membrane feeder system (Coelen Glastechniek, Weldaad, Netherlands). All surviving mosquitoes were dissected on the seventh day after the feeding assay; midguts were stained in 1% mercurochrome and examined for the presence and density of oocysts by expert microscopists.

### Data analysis

Data analysis was conducted using R (R foundation for statistical computing, Vienna, Austria; v3.6.3) [20]. Asexual parasite and gametocyte density data were compared between groups by Wilcoxon signed rank-sum test, and comparisons between gametocyte prevalence between group and time-point were conducted by Fisher’s exact test. Quantified HRP-2 values that fell below the lower limit of quantification (LLOQ) were treated as LLOQ/2. For the analysis of HRP-2 prevalence, HRP-2 positivity was defined as any quantity of HRP-2 greater than the mean plus 3*SD of HRP-2 in individuals treated with ACT + PQ at day 49 post-treatment (n = 45). Multivariate survival analysis of time to RDT and HRP-2 negativity between groups was conducted by Cox proportional hazards regression. Differences in quantified HRP-2 levels between groups over time were compared by linear regression modelling and differences between mosquito infectious and non-infectious individuals were compared by logistic regression.

## Results

Participants were categorised according to artemisinin treatment with (ACT + PQ) or without (ACT-only) the gametocytocidal drug primaquine to allow for the evaluation of the contribution of gametocytes to HRP-2 levels and RDT positivity. As such, at baseline each treatment group consisted of 50 individuals. At enrolment, median asexual stage parasite density by qRT-PCR was greater in the ACT + PQ group (Additional file 1: Fig. S1**)**, though not significantly so (Wilcoxon rank-sum, p = 0.26) and gametocyte densities were similar between groups (Table 1).

**Table 1:** Baseline sample characteristics

Treatment group	N	Female, n (%)	Age, median (range)	Baseline asexual density by qRT-PCR, median (IQR)	Baseline gametocyte density by qRT-PCR, median (IQR)
ACT	50	21 (42)	11.5 (5–40)	289.3 (29.4–1724.5)	77.3 (37.1–124.0)
ACT + PQ	50	31 (62)	10 (5–47)	692.4 (55.1–4247.6)	60.3 (24.8–175.9)

**Fig. 1:**
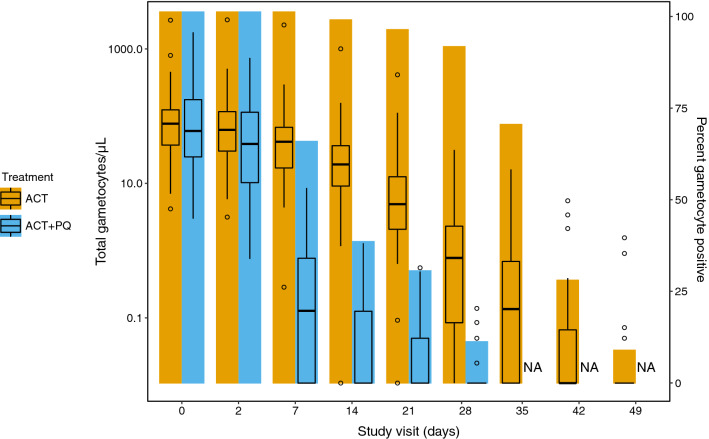
Gametocyte density and positivity at each study visit. Boxes represent gametocyte density (left axis) measured by qRT-PCR, presented as median and interquartile range. Bars represent percentage gametocyte positivity (right axis)

Asexual parasite density fell rapidly after initial treatment, with median asexual density reaching zero in both groups by day 7 (p = 0.57), remaining as such for the duration of the study (Additional file 1: Fig. S1). Whilst gametocyte density declined over the full study period in both groups, gametocyte clearance occurred much more rapidly in individuals treated with primaquine. A statistically significant decrease in gametocyte density was observed by day 2 in the ACT + PQ group (Wilcoxon rank-sum, p = 0.05) whilst such a decrease was not observed in the ACT-only group until day 7 (p = 0.003). By day 7, gametocyte positivity had fallen to 65.2% in the ACT + PQ treatment group but remained at 100% in the ACT-only group (Table 2, Fig. 1). Median gametocyte density fell to 0 by day 14 in the ACT + PQ group, but took until day 42 to drop to the same density in the ACT-only group. A far greater number of individuals remained gametocyte positive in the ACT-only group (90.7%) compared to the ACT + PQ group (11.4%) at day 28 (Fisher’s exact test, p =  < 0.0001) (Table 2, Fig. 1).

**Fig. 2:**
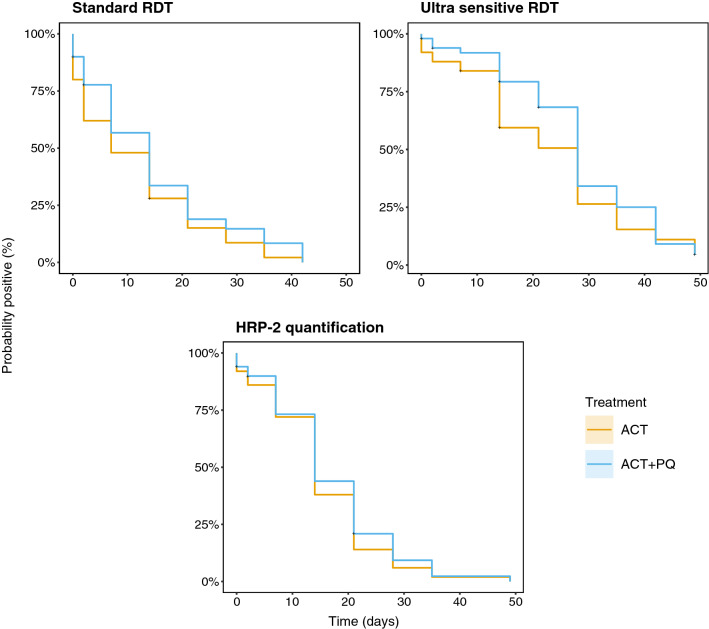
Kaplan–Meier plots showing time to negativity by standard RDT, ultra-sensitive RDT and quantified HRP-2 between the ACT group (orange) and the ACT + PQ group (blue)

Survival analysis of test positivity by standard RDT, uRDT and HRP-2 quantification shows that the longest duration of test positivity was with uRDT then HRP-2 quantification and standard RDT respectively (Fig. 2). Treatment with primaquine did not significantly influence the time to test negativity by uRDT (Cox proportional hazards ratio (HR) 0.79 [95% CI 0.52–1.21], p = 0.28), RDT (HR 0.77 [95% CI 0.51–1.15], p = 0.20) or HRP-2 (HR 0.88 [95% CI 0.59–1.32], p = 0.53), though the observed trend is suggestive of a slight delay in time to negativity in the ACT-PQ group relative to the ACT-only group–likely an effect of greater asexual parasite density at baseline leading to increased HRP-2 antigenaemia (Additional file 1: Fig. S2). However, no effect on time to test negativity was found when adjusting for baseline asexual density with any of the three tests: uRDT, HR 0.82 [95% CI 0.53–1.26], p = 0.36; HRP-2, HR 0.93 [95% CI 0.61–1.41], p = 0.727; and RDT, HR 0.83 [95% CI 0.55–1.26], p = 0.39.

**Fig. 3:**
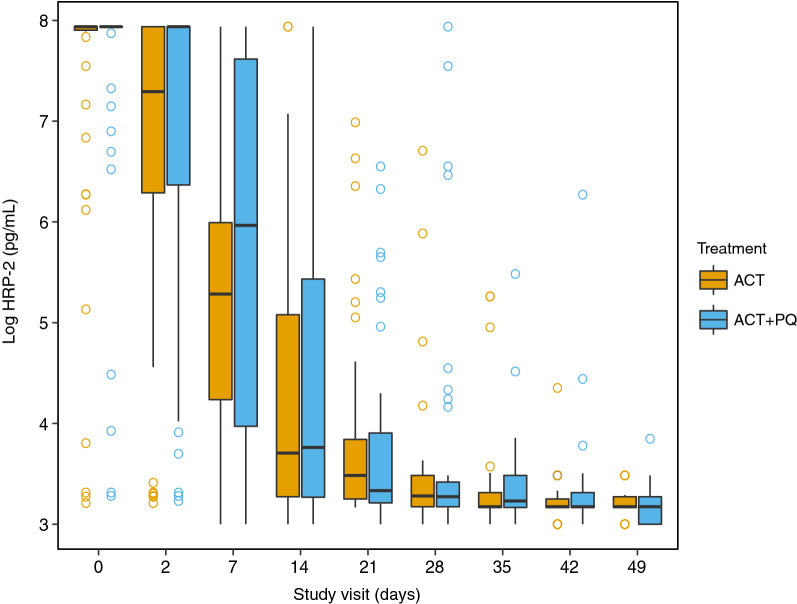
HRP-2 levels at each study visit by treatment group. Boxes are presented as median and interquartile range. For clarity of presentation in this figure only, individuals with HRP-2 levels lower than the lower limit of quantification (LLOQ) have been inflated to the LLOQ

Quantified levels of HRP-2 were similar between groups at all time-points, though a tendency towards an increased concentration was observed in the ACT + PQ group, in line with greater asexual parasite densities at baseline (Fig. 3). Linear regressions revealed no statistically significant difference between treatment groups, nor were any differences observed when adjusting for baseline asexual density (Table 3). Levels of HRP-2 declined over 14 days post-treatment, with HRP-2 positivity falling from 92% to 42.9% and 94% to 45.8% in the ACT-only and ACT + PQ groups respectively (Table 3). By day 21, variability in quantified HRP-2 levels between treatment groups was negligible and most individuals were approaching the lower limit of detection of the assay (Table 3). HRP-2 positivity continued to decrease over time until all individuals were found to be negative according to the assay cut-off by day 49.

**Table 2:** Parasite densities by study visit

Study visit (days)	Treatment group	N	Asexual density by qRT-PCR, median (IQR)	Gametocyte density by qRT-PCR, median (IQR)
0	ACT	50	289.3 (29.4–1724.5)	77.3 (37.1–124.0)
	ACT + PQ	50	692.4 (55.1–4247.6)	60.3 (24.8–175.9)
2	ACT	50	0 (0–1.2)	62.6 (30.23–116.5)
	ACT + PQ	49	0.2 (0–1.5)	38.6 (10.3–114.2)
7	ACT	50	0 (0–0.1)	41.6 (17.0–68.2)
	ACT + PQ	48	0 (0–0.2)	0.1 (0–0.8)
14	ACT	49	0 (0)	19.2 (9.2–36.4)
	ACT + PQ	48	0 (0–0.1)	0 (0–0.1)
21	ACT	44	0 (0)	4.9 (2.1–12.7)
	ACT + PQ	46	0 (0)	0 (0)
28	ACT	45	0 (0)	0.8 (0.1–2.3)
	ACT + PQ	45	0 (0)	0 (0)
35	ACT	45	0 (0)	0.1 (0–0.7)
	ACT + PQ	45	NA	NA
42	ACT	44	0 (0)	0 (0–0.1)
	ACT + PQ	45	NA	NA
49	ACT	44	0 (0)	0 (0)
	ACT + PQ	45	NA	NA

**Table 3:** Test positivity and HRP-2 levels by study visit

	Treatment group	N	RDT positve (%)	Ultra sensitive RDT positive (%)	HRP-2 positive (%)	HRP-2 concentration (pg/mL), median (IQR)	p-value	Adjusted p-value
0	ACT	50	40 (80)	46 (92)	46 (92)	> 2800 (2704.3– > 2800)	0.597	0.944
	ACT + PQ	50	45 (90)	49 (98)	47 (94)	> 2800 (> 2800– > 2800)		
2	ACT	50	31 (62)	44 (88)	41 (82)	1469.6 (538.2– > 2800)	0.375	0.679
	ACT + PQ	49	39 (79.69)	46 (93.9)	44 (89.8)	> 2800 (582.2– > 2800)		
7	ACT	50	26 (52)	42 (84)	38 (76)	197.3 (69.3–400.8)	0.210	0.453
	ACT + PQ	48	30 (62.5)	44 (91.7)	35 (72.9)	393.3 (53.1–2056.2)		
14	ACT	49	15 (30.6)	29 (59.2)	21 (42.9)	40.7 (26.4–1600.6)	0.552	0.957
	ACT + PQ	48	21 (43.8)	38 (79.2)	22 (45.8)	43.05 (26.3–229.6)		
21	ACT	44	9 (20.5%)	22 (50)	9 (20.5)	32.6 (25.8–46.6)	0.871	0.577
	ACT + PQ	46	15 (32.69%)	32 (69.6)	11 (23.9)	28.05 (24.8–49.7)		
28	ACT	45	5 (11.1%)	12 (26.7)	4 (8.9)	26.6 (23.9–32.6)	0.503	0.479
	ACT + PQ	45	9 (20%)	15 (33.3)	8 (17.8)	26.4 (23.9–30.5)		
35	ACT	45	2 (4.4%)	7 (15.6)	3 (6.7)	23.9 (23.7–27.5)	0.217	0.154
	ACT + PQ	45	5 (11.1%)	12 (26.7)	2 (4.4)	25.3 (23.7–32.6)		
42	ACT	44	0 (0%)	5 (11.4)	1 (2.3)	23.9 (23.7–25.8)	0.419	0.414
	ACT + PQ	45	0 (0%)	5 (11.1)	2 (4.4)	23.9 (23.7–27.5)		
49	ACT	44	0 (0%)	2 (4.5)	0 (0)	23.9 (23.7–26.4)	0.253	0.365
	ACT + PQ	45	0 (0%)	5 (11.1)	0 (0)	23.9 (< 6.8–26.4)		

**Fig. 4:**
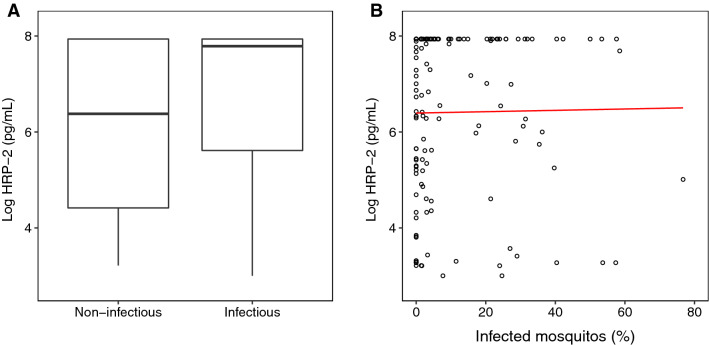
Levels of HRP-2 compared **A** between mosquito infectious and non-infectious individuals on days 0, 2 and 7 in the ACT group (boxes represent median and interquartile range), and **B** against the percentage of mosquitoes infected during feeding in mosquito infectious individuals (red line represents fitted linear regression model)

Mosquito infectivity was analysed at day 0, 2 and 7 within the ACT-only group. HRP-2 levels were not significantly different between mosquito infectious and non-infectious individuals (Odds ratio (OR) 1.19 [95% CI 0.98–1.46], p = 0.077), nor was any significant difference observed when adjusting for baseline gametocyte density and day of study visit (OR 1.23 [95% CI 0.96–1.59], p = 0.12) (Fig. 4A**)**. Looking at only individuals who were infectious to mosquitoes, linear regression showed no significant relationship between the percentage of mosquitoes infected and levels of HRP-2 with (p = 0.31) or without adjustment for baseline gametocyte density and study visit day (p = 0.83) (Fig. 4B). Finally, when comparing time to test negativity, no significant difference was found between mosquito infectious and non-infectious individuals with and without adjustment for baseline gametocyte density (Additional files 1: Fig. S3, Table S1).

## Discussion

Malaria diagnosis by HRP-2-based rapid diagnostic tests is widespread. Prior to this study, it was unknown if gametocytes contribute to the persistence of HRP-2 after ACT. Understanding how gametocytes may contribute to HRP-2 levels and RDT positivity would improve interpretation of RDTs and uRDTs. If gametocytes are detectable after standard ACT via HRP-2-based diagnostics, it will also be important to establish the infectivity of such individuals. In this study we have evaluated the contribution of sexual stage parasites to RDT and uRDT positivity, and HRP-2 levels over time in malaria-infected, gametocyte positive individuals treated with or without the gametocytocidal drug primaquine. As expected, gametocytes were rapidly cleared after PQ treatment but no significant differences in time to negativity by standard RDT, uRDT or quantified HRP-2 was observed. Correspondingly, levels of HRP-2 were found to be similar between the two treatment groups, with any differences likely attributable to baseline asexual parasite densities. These results address the concern that the lower limits of detection afforded by HRP-2-based uRDTs may lead to false-positive test results in individuals who do not harbour replicating blood-stage infection, but have not yet cleared gametocytes from circulation [21, 22]. These data suggest that surviving gametocytes after ACT do not significantly contribute to HRP-2 load.

The lag between parasite and HRP-2 clearance after standard ACT treatment has led to concerns that RDT results may often be confounded [21, 23]. Evidence suggests that HRP-2 is actively produced and secreted by asexual and early sexual stage parasites, and is internalised by mature gametocytes – though it is unclear when transcription and translation ceases [6, 7]. HRP-2 has been proposed to play a role in the metabolism of haem, and also in the facilitation of cytoadherence [24]; a lack of active production by mature gametocytes is thus fitting for what are metabolically quiescent, circulating parasites, and this has led to a lack of concern that mature gametocytes are of significance in the context of persistent HRP-2 antigenaemia post-treatment. However, the evidence for the relationship between gametocytes and HRP-2 antigenaemia is currently conflicting and sparse. Residual sexual stage parasites have been shown to be both associated [25] and unassociated [26] with RDT positivity, though such studies lack sufficiently sensitive experimental approaches to drawn firm conclusions. In light of the dropping threshold for HRP-2 detection in the context of uRDTs, it is important to clarify unambiguously whether persistent gametocytes following treatment contribute to false test positivity.

Multivariate analysis found that the primary factor associated with initial HRP-2 levels and subsequent decay was asexual parasitaemia at enrolment in the study, as has been demonstrated previously [27, 28]. Although most blood stages are known secrete HRP-2, asexual parasites by far constitute the greatest proportion of total parasitaemia, thus it follows that infections with higher asexual densities produce higher levels of HRP-2. The persistence of HRP-2 after ACT has been shown to be caused by the return of previously infected erythrocytes (containing exported HRP-2) to circulation after the physical removal of dead asexual parasites [29]. That residual gametocytes do not significantly contribute to HRP-2 levels after treatment with ACT in this study will facilitate use and interpretation of more sensitive diagnostics based on detection of HRP-2, however it will be important to further examine whether this relationship is maintained across varying transmission settings and between genetic HRP-2 variant parasites, where gametocyte densities may be higher or variants may produce greater levels of HRP-2.

When comparing time to negativity between the standard RDT and uRDT, results were as anticipated, with individuals testing negative sooner by RDT than uRDT due to lower sensitivity. Unexpectedly, time to negativity based on quantified levels of HRP-2 was more similar to RDT than uRDT. This is likely due to the calculated cut-off of 54 pg/mL used to determine positivity by quantified HRP-2 versus the visual confirmation of the appearance of any line on the uRDT, which is sensitive down to 10–40 pg/mL [9]. The cut-off used in this study was calculated based on HRP-2 levels in individuals who were treated, and confirmed as continuously parasite negative by molecular methods over 49 days. As such, while the threshold for true HRP-2 negativity by this method may be less stringent, it is likely more representative of natural infections. Practically this finding supports a rationale for the more conservative use of RDTs post-treatment and highlights the importance of parasitological diagnosis when assessing potential treatment failure or reinfection. Within a research context, the findings of this study support calls for the use of additional methods based on antigen quantification to differentiate active infection from residual antigenaemia; models based on the quantified decay and ratio of HRP-2 to p-LDH have shown an accuracy of 77.5% in distinguishing patients with and without current infection [30]. Future development in methods to quantify *Plasmodium* antigens in a lateral flow device format may also prove to be useful as a research tool. Such approaches are already in the early stages of application for diseases such as prostate cancer, whereby a portable reader is able to measure the intensity of the test line, and thus quantity of prostate-specific antigen [31]. This type of innovation has the potential to be beneficial from a research perspective, quickly and easily providing epidemiological data to help direct RDT use–particularly in low transmission settings [32].

A difficulty in the context of such antigen quantification is accurately capturing the wide range of physiological concentrations observed during infection. In this study, HRP-2 levels were measured in samples diluted to 1:10, and yet signal exceeding the upper limit of quantification was observed in many samples–particularly at day 0 and 2. However, by increasing the dilution of samples to reduce saturation at the upper limit, the lower limit of quantification within a sample is correspondingly increased, leading to loss of data at the lower end of the quantifiable range. Whilst the issue of sample dilution will be highly relevant irrespective of the assay platform used, other approaches may provide greater levels of flexibility compared to commercial kits. Suspension bead array-based techniques have already been developed for the detection of HRP-2 and p-LDH [27, 28] and allow finer control of parameters such as the concentration of capture antibody used or the range of standard curve used to quantify the analyte.

In summary, this study has shown that the presence of gametocytes post antimalarial treatment appears to have little impact on whether HRP-2 based RDT test positive or measurable levels of circulating HRP-2. These data are consistent with other studies suggesting that asexual parasites are the primary source of HRP-2 which is perhaps unsurprising considering these parasites are multiplicative and present at markedly higher densities than gametocytes. These data provide clarity on the interpretation of RDT results and HRP-2 levels for future clinical and epidemiological studies.

## Conclusions

The results of this study indicate that surviving sexual stage parasites after treatment do not affect time to negativity by standard or ultra-sensitive RDT, or levels of circulating HRP-2. In addition, the findings presented here support previous data showing that asexual parasites are the main source of HRP-2 during infection, and that initial asexual parasite density primarily determines how long an individual will test positive by RDT. Importantly, this study provides evidence to support the use of ultra-sensitive RDTs in diagnosis and surveillance in spite of persisting sexual stage parasites following standard malaria treatment.

## Supplementary Information

**Additional file 1: Figure S1.** Asexual parasite densities at each study visit by treatment group, as measured by qPCR. Boxes are presented as median and interquartile range. **Figure S2.** Comparison of levels of HRP-2 between ACT and ACT+PQ group stratified by baseline asexual density. Solid black line represents mean levels of HRP-2. Dotted black lines at study visit 7 are to assist in identifying differences in mean levels of HRP-2 between groups and strata. **Figure S3.** Kaplan-Meier plots showing time to negativity by standard RDT, ultra-sensitive RDT and quantified HRP-2 between the mosquito non-infectious (green) and mosquito infectious (red) individuals. **Table S1.** Cox proportional hazards ratio by test, between infectious and non infectious individuals. Reported with and without adjustment for baseline gametocyte density.

## Data Availability

Data are available upon reasonable request to the corresponding author.

## Abbreviations

ACT: Artemisinin-based combination therapy
DP: Dihydroartemisinin-piperaquine
ELISA: Enzyme linked immunosorbent assay
HRP-2: Histidine-rich protein 2
LLOQ: Lower limit of quantification
PA: Pyronaridine-artesunate
PQ: Primaquine
qRT-PCR: Quantitative real-time polymerase chain reaction
RDT: Rapid diagnostic test
uRDT: Ultra-sensitive rapid diagnostic test
WHO: World Health Organization
