# Supplementary material for: Persistence of Plasmodium falciparum HRP-2 antigenaemia after artemisinin combination therapy is not associated with gametocytes
Source: Malar J. 2022 Dec 6;21:372. doi: 10.1186/s12936-022-04387-0 (PMC9724264; doi:10.1186/s12936-022-04387-0)
Supplement: Supplementary file 1 — Additional file 1: Figure S1. Asexual parasite densities at each study visit by treatment group, as measured by qPCR. Boxes are presented as median and interquartile range. Figure S2. Comparison of levels of HRP-2 between ACT and ACT+PQ group stratified by baseline asexual density. Solid black line represents mean levels of HRP-2. Dotted black lines at study visit 7 are to assist in identifying differences in mean levels of HRP-2 between groups and strata. Figure S3. Kaplan-Meier plots showing time to negativity by standard RDT, ultra-sensitive RDT and quantified HRP-2 between the mosquito non-infectious (green) and mosquito infectious (red) individuals. Table S1. Cox proportional hazards ratio by test, between infectious and non infectious individuals. Reported with and without adjustment for baseline gametocyte density. [file 12936_2022_4387_MOESM1_ESM.pdf]

Table of Contents

Additional Figure S1 1

Additional Figure S2 2

Additional Figure S3 3

Additional Table S1 4

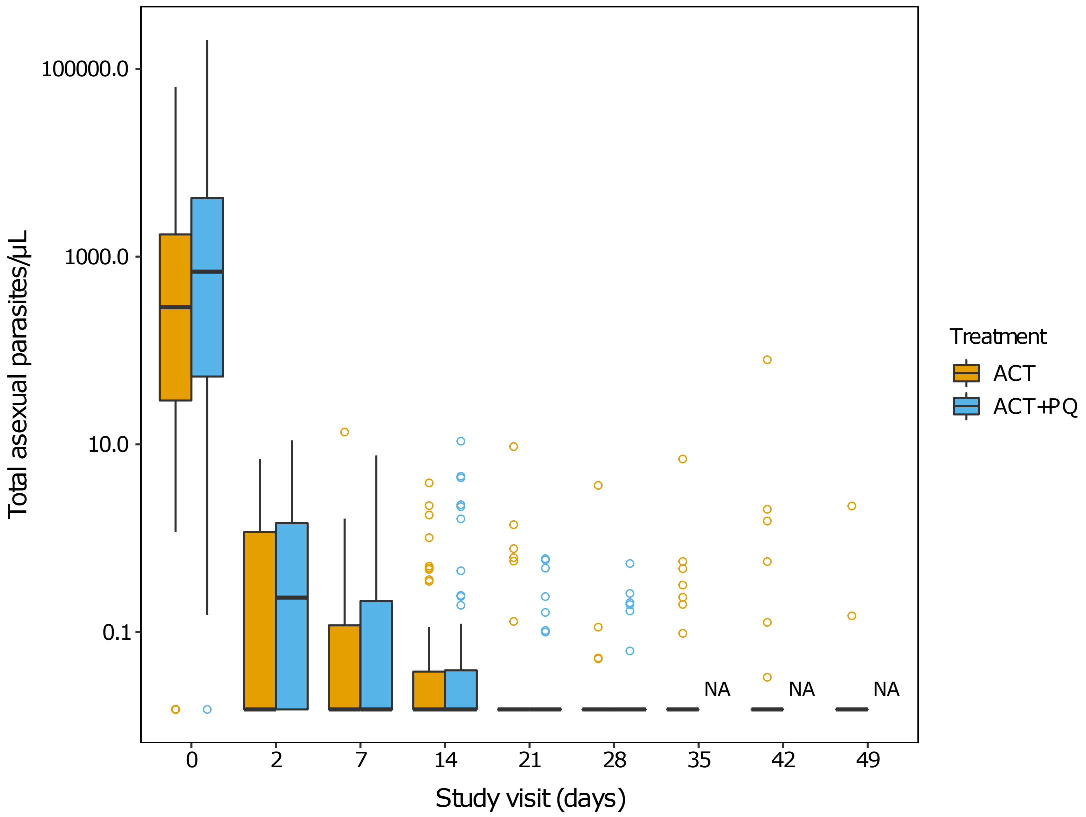

Additional Figure S1. Asexual parasite densities at each study visit by treatment group, as measured by qPCR. Boxes are presented as median and interquartile range.

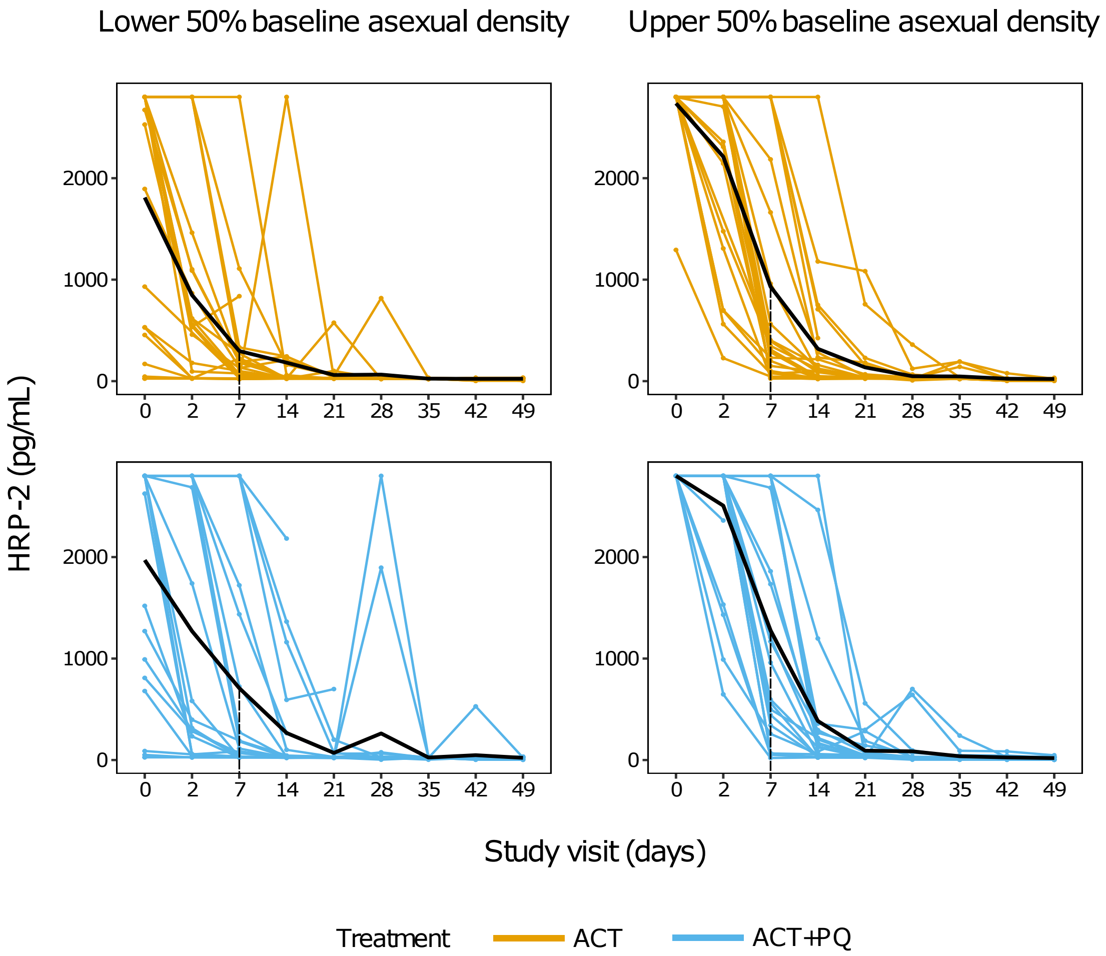

Additional Figure S2. Comparison of levels of HRP-2 between ACT and ACT+PQ group stratified by baseline asexual density. Solid black line represents mean levels of HRP-2. Dotted black lines at study visit 7 are to assist in identifying differences in mean levels of HRP-2 between groups and strata.

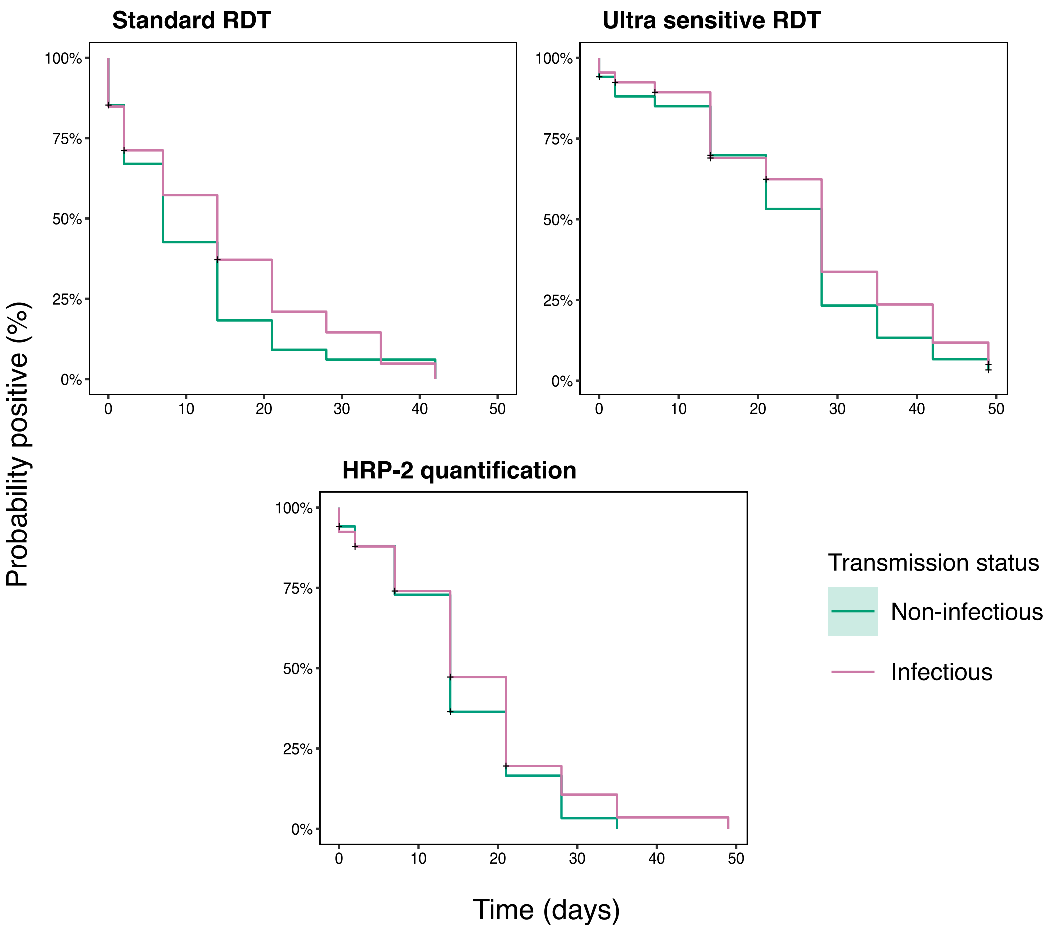

Additional Figure S3 . Kaplan-Meier plots showing time to negativity by standard RDT, ultra-sensitive RDT and quantified HRP-2 between the mosquito non-infectious (green) and mosquito infectious (red) individuals.

Supplementary table 1. Cox proportional hazards ratio by test, between infectious and non-infectious individuals. Reported with and without adjustment for baseline gametocyte density.

Test	Variate	Coefficients	Hazard ratio	95% CI	p
Standard RDT	Univariate	Infectious at day 0	0.76	0.49-1.16	0.197
	Multivariate	Infectious at day 0	0.76	0.49-1.18	0.229
		Baseline gametocyte density	1	1	0.763
Ultra sensitive RDT	Univariate	Infectious at day 0	0.79	0.51-1.24	0.309
	Multivariate	Infectious at day 0	0.80	0.50-1.27	0.344
		Baseline gametocyte density	1	1	0.607
Quantified HRP-2	Univariate	Infectious at day 0	0.79	0.51-1.22	0.286
	Multivariate	Infectious at day 0	0.79	0.50-1.24	0.301
		Baseline gametocyte density	1	1	0.677
